# New species of *Pseudopoda* Jäger, 2000 from Southern China (Araneae, Sparassidae)

**DOI:** 10.3897/zookeys.361.6089

**Published:** 2013-12-12

**Authors:** Feng Zhang, Bao-Shi Zhang, Zhi-Sheng Zhang

**Affiliations:** 1 The Key Laboratory of Invertebrate Systematics and Application, College of Life Sciences, Hebei University, Baoding, Hebei 071002, P. R. China; 2 Department of Biochemistry, Baoding University, Baoding, 071051, P. R. China; 3 Key Laboratory of Eco-environments in Three Gorges Reservoir Region, School of Life Science, Southwest University, Chongqing 400715, China

**Keywords:** Taxonomy, Heteropodinae, huntsman spiders, new species

## Abstract

Four new species of the huntsman spider genus *Pseudopoda* Jäger, 2000 are described from Southern China: *Pseudopoda acuminata* **sp. n.** from Suiyang County, Guizhou Province, *P. emei* **sp. n.** from Emei Mountain, Sichuan Province, *P. lacrimosa* **sp. n.** from Fugong County and Tengchong County, Yunnan Province, and *P. robusta* **sp. n.** fromJinyun Mountain, Chongqing Municipality.

## Introduction

The members of the huntsman spider family Sparassidae Bertkau, 1872 are small to large sized spiders. Currently it includes 84 genera and 1132 known species (Platnick 2013). Of these, 102 species from eleven genera (*Bhutaniella* Jäger, 2000, *Eusparassus* Simon, 1903, *Gnathopalystes* Rainbow, 1899, *Heteropoda* Latreille, 1804, *Micrommata* Latreille, 1804, *Olios* Walckenaer, 1837, *Pseudopoda* Jäger, 2000, *Rhitymna* Simon, 1897, *Sagellula* Strand, 1942, *Sinopoda* Jäger, 1999 and *Thelcticopis* Karsch, 1884) have been recorded from China (Song et al. 1999; Platnick 2013).

The genus *Pseudopoda*, established by Jäger (2000), belongs to the subfamily Heteropodinae Thorell, 1873. After that, Jäger (2001) made a major revision on Himalayan representatives and described 51 new species, for which he proposed five species groups. Jäger and Vedel (2007) revised the genus *Pseudopoda* of Yunnan Province, China and described 15 new species, they discussed species groups proposed by Jäger (2001) with respect to the new results of their study. Several papers with descriptions of species from Asia have been published. Seven new species from India (Jäger 2001, 2002, 2008a), five new species from Laos (Jäger 2007; Jäger et al. 2006; Jäger and Praxaysombath 2009), two new species from Japan (Jäger and Ono 2002; Ono 2009;) and one new species from Vietnam (Jäger and Vedel 2005), have been described. Several papers also included transfers, twelve taxa were transferred from *Heteropoda* to *Pseudopoda* (Jäger 2000, 2001, 2002; Jäger and Yin 2001) and two taxa from *Sinopoda* to *Pseudopoda* (Jäger 2001; Jäger and Vedel 2007). To date, 98 species of the genus *Pseudopoda* have been recorded from Asia, of which 37 have been recorded from China (Jäger 2008b; Jäger and Ono 2001; Jäger et al. 2002; Liu et al. 2008; Sun and Zhang 2012; Tang and Yin 2000; Xu and Yin 2000; Yang and Chen 2008; Yang and Hu 2001; Yang et al. 2009; Zhang and Kim 1996).

During the examination of spider specimens collected from Southern China, four new species, *Pseudopoda acuminata* sp. n., *Pseudopoda emei* sp. n., *Pseudopoda lacrimosa* sp. n. and *Pseudopoda robusta* sp. n. were recognized and are here described. The systematic position of the new species within the genus is discussed. A distribution map of the new *Pseudopoda* species in China is also provided.

## Material and methods

All specimens were kept in 75% ethanol and examined, drawn and measured under a Nikon SMZ1500 stereomicroscope, equipped with a camera lucida. Photos were taken with a Leica M205A stereomicroscope equipped with a DFC450 CCD camera. Measurement system mostly follows Jäger (2000). Carapace length was measured from the anterior margin to the rear margin of the carapace medially in dorsal view. Two eye rows were described in dorsal view. Eye sizes were measured as the maximum diameter of the lens in dorsal or frontal view. The measurements of legs are shown as: total length (femur, patella, tibia, metatarsus, tarsus). Spine notation follows Davies (1994). Spines are listed for each segment in the following order: prolateral, dorsal, retrolateral, ventral; a three digit formula indicates ventral spines missing. The epigyne was cleared in a warm solution of potassium hydroxide (KOH), transferred to 75% ethanol for drawing. All measurements are in millimeters. All specimens studied are deposited in the Museum of Hebei University (MHBU, curator: Prof. Guodong Ren), Baoding, China and School of Life Science, Southwest University, Chongqing, China (SWUC, curator: Prof. Zhisheng Zhang).

### Abbreviations

AB anterior longitudinal bands; aEF anterior margin of epigynal field; ALE anterior lateral eyes; AME anterior median eyes; amLL anterior margin of lateral lobes; C conductor; CD copulatory duct; CQ Chongqing Municipality, China; E embolus; EP embolic projection; FD fertilization duct; GLGS Gaoligongshan; KKS Kuankuoshui Nature Reserve; LL lateral lobes of epigyne; lmLL lateral margin of lateral lobes; MF median field of epigyne; mmLL median margin of lateral lobes; MOA median ocular area; PI posterior incisions; PLE posterior lateral eyes; pmLL posterior margins of lateral lobes; PME posterior median eyes; R ridges; RTA retrolateral tibial apophysis; S spermathecae; SC Sichuan Province, China; SD sperm duct; SP
Sparassidae; ST subtegulum; T tegulum.

## Taxonomy

### Sparassidae Bertkau, 1872
Heteropodinae Thorell, 1873

#### Pseudopoda

Jäger, 2000

http://species-id.net/wiki/Pseudopoda

##### Type species.

*Sarotes promptus* O. P.-Cambridge, 1885

##### Diagnosis.

Conductor of male palp membranous; embolus broadened and flattened or at least in its proximal part broadened; retrolateral tibial apophysis arising in a medial or basal position; lateral lobes of epigyne rising beyond epigastric furrow, and covering median septum (Jäger 2000, 2001).

##### Distribution.

China, Nepal, Bhutan, Myanmar, Thailand, Vietnam, Laos, Pakistan and India.

#### Pseudopoda acuminata sp. n.

http://zoobank.org/B85A9DF9-D168-4542-A80E-B15667209820

http://species-id.net/wiki/Pseudopoda_acuminata

Figs 1 –17

##### Type material.

**Holotype** ♂ (SP–KKS–10–0816), from CHINA: Guizhou Province, Suiyang County, Kuankuoshui Nature Reserve (28°17'N, 107°11'E, 1200 m), 16. VIII.2010, Z.S. Zhang leg. (hand collecting), deposited in SWUC. **Paratype:** 1♀ (SP–KKS–10–0817), same data as holotype.

##### Etymology.

The specific name is derived from the Latin word ‘acuminatus, -a, -um’, meaning ‘acuminate’, referring to the acute shape of the embolic projection; adjective.

##### Diagnosis.

Male and female of *Pseudopoda acuminata* sp. n. resemble those of *Pseudopoda contentio* Jäger & Vedel, 2007 by: embolus sickle-shaped and bent in a semicircle, embolic projection small, anterior margins of lateral lobes diagonal, internal duct system visible through cuticle in a ventral view as large, rather elongated patches. They are distinguished from the latter species by the following combination of characters: embolic projection spine-shaped (Figs 4, 11, 13); dorsal branch of retrolateral tibial apophysis slightly curved, ventral branch as a small hump (Figs 4–5, 11–12); anterior rims of lateral lobes curved, running more diagonal and pointing 30° anterio-laterally (Figs 8, 14); extending part of lateral lobes more narrow in dorsal view (Figs 9, 15); posterior end of first winding of internal duct system covered by lateral lobes (Figs 9, 15).

**Figures 1–4.**
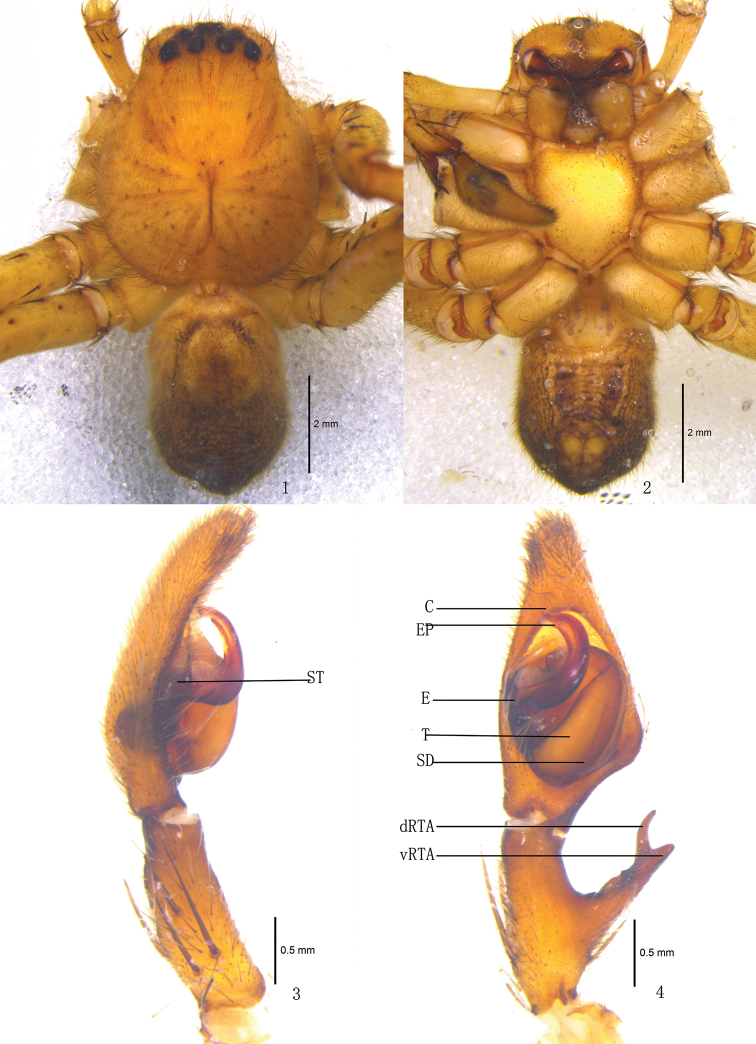
*Pseudopoda acuminata* sp. n., Male (SP–SC–03–0050): **1–2** Body (**1** dorsal **2** ventral) **3–4** Left palp (**3** prolateral **4** ventral). Abbreviations: C conductor; dRTA dorsal branch of retrolateral tibial apophysis; E embolus; EP embolic projection; SD sperm duct; ST subtegulum; T tegulum; vRTA ventral branch of retrolateral tibial apophysis. Scale bars: 2 mm (**1–2**); 0.5 mm (**3–4**).

**Figures 5–9.**
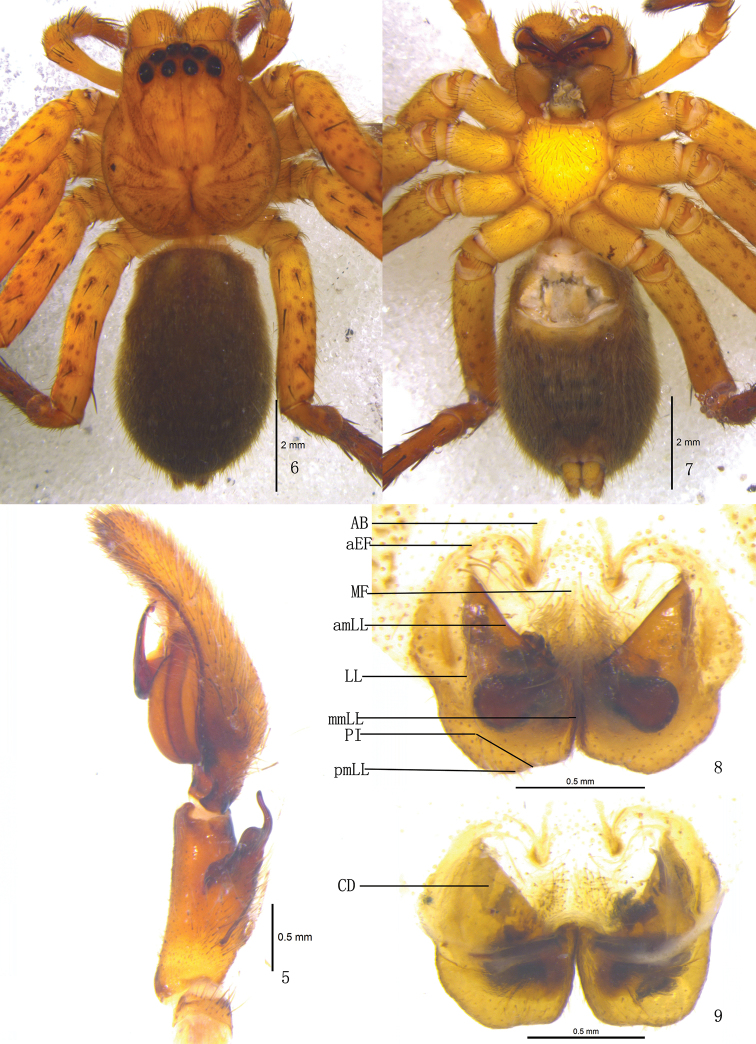
*Pseudopoda acuminata* sp. n., **5** Left palp of male (retrolateral). **6–9** Female (SP–SC–03–0052): **6–7** Body (**6** dorsal **7** ventral) **8–9** Epigyne (**8** ventral **9** dorsal). Abbreviations: AB anterior bands; aEF margin of epigynal field; amLL anterior margin of lateral lobes; CD copulatory duct; LL lateral lobes of epigyne; MF median field of epigyne; mmLL median margin of lateral lobes; pmLL posterior margins of lateral lobes; PI posterior incisions. Scale bars: 2 mm (**6–7**); 1 mm (**5**, **8–9**).

**Figures 10–17.**
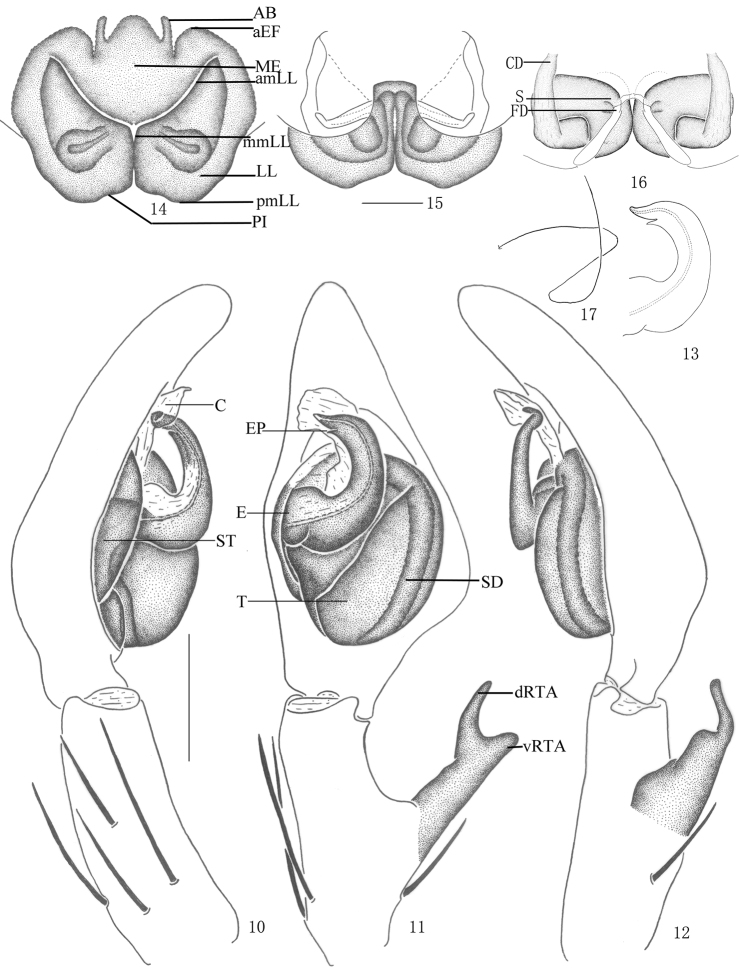
*Pseudopoda acuminata* sp. n., **10–13** Male (SP–SC–03–0050): **10–12** Left palp (**10** prolateral **11** ventral **12** retrolateral) **13** embolus (ventral) **14–17** Female (SP–SC–03–0052): **14–16** Epigyne (**14** ventral **15** dorsal **16** apical); **17** Schematic course of internal duct system, dorsal. Abbreviations: AB anterior bands; aEF anterior margin of epigynal field; amLL anterior margin of lateral lobes; C conductor; CD copulatory duct; dRTA dorsal branch of retrolateral tibial apophysis; E embolus; EP embolic projection; FD fertilization duct; LL lateral lobes of epigyne; MF median field of epigyne; mmLL median margin of lateral lobes; pmLL posterior margins of lateral lobes; PI posterior incisions; S spermathecae; SD sperm duct; ST subtegulum; T tegulum; vRTA ventral branch of retrolateral tibial apophysis. Scale bars: 0.5 mm.

##### Description.

Holotype (SP–KKS–10–0816): total length 11.23; prosoma 6.61 long, 4.82 wide; opisthosoma 4.58 long, 2.83 wide. Prosoma with some setae. Fovea long, longitudinal. Coloration: Dorsal shield of prosoma yellow brown. Radial furrows brownish. Fovea dark brown. Ocular area yellowish brown. Chelicerae yellowish brown. Labium, gnathocoxae and sternum yellow. Legs yellow, with dark dots randomly distributed, and especially on the setae and spine bases of coxa and femur. Opisthosoma color overall dark brown (Fig. 1), yellowish anterior-dorsally, ventral median dark brown (Fig. 2). Both eye rows slightly recurved. Eye diameters and interdistances: AME 0.26, ALE 0.36, PME 0.27, PLE 0.36; AME–AME 0.29, AME–ALE 0.05, PME–PME 0.42, PME–PLE 0.49. MOA 0.87 long, anterior width 0.60, posterior width 0.87. Clypeus height 0.26. Chelicerae with three promarginal and four retromarginal teeth, and with thirteen denticles between them. Sternum with dark setae. Leg measurements: I 24.25 (6.21, 2.10, 6.82, 6.52, 2.60), II 24.44 (6.23, 2.14, 6.88, 6.55, 2.64), III 23.79 (6.13, 1.96, 6.89, 6.35, 2.37), IV 24.12 (6.18, 2.03, 6.82, 6.52, 2.57). Leg formula: 2143. Leg spination: palps 131, 101, 2121; femur I–II 323, III 322, IV 331; patella I–III 101, IV 001; tibia I–II 2226, III–IV 2126; metatarsus I–II 2024, III 2026, IV 3036. Male palp. Embolus sickle-shaped, arising from 9- to 10-o’clock-position on tegulum, embolic tip pointing prolaterally (Figs 3–4, 10–11); sperm duct running submarginally along retrolateral margin of tegulum in ventral view (Figs 4–5, 11–12); EP spine-shaped (Figs 4, 11, 13); RTA long, with broad base, arising medially from tibia, dorsal branch long and thin, slightly curved, ventral branch short and thick, with blunt tip (Figs 4–5, 11–12).

Female. Paratype (SP–KKS–10–0817): total length 9.70; prosoma 4.51 long, 3.02 wide; opisthosoma 5.22 long, 2.82 wide. Coloration: Dorsal shield of prosoma reddish brown. Legs yellowish brown, with dark dots randomly distributed, and especially on the setae and spine bases of coxa and femur. Opisthosoma color overall dark brown (Figs 6–7). Eye diameters and interdistances: AME 0.21, ALE 0.32, PME 0.23, PLE 0.31; AME–AME 0.21, AME–ALE 0.13, PME–PME 0.36, PME–PLE 0.44. MOA 0.82 long, anterior width 0.60, posterior width 0.83. Clypeus height 0.25. Leg measurements: I 15.47 (4.62, 1.83, 3.81, 3.91, 1.30), II 15.72 (4.66, 1.83, 3.98, 3.93, 1.32), III 14.78 (4.55, 1.77, 3.62, 3.65, 1.19), IV 15.31 (4.59, 1.85, 3.75, 3.82, 1.30). Leg formula: 2143. Leg spination: palps 131, 101, 2121, 2112; femur I–II 323, III 322, IV 331; patella I–IV 001; tibia I 2026, II–IV 2126; metatarsus I–II 2024, III 2026, IV 3036. Epigyne. Epigynal field wider than long, anterior margin rather indistinct, anterior longitudinal bands thin and short (Figs 8, 14); LLs wider at the median part, touching each other along the median line, anterior margin of LLs pointing 30° anterior-laterally; posterior margins of LLs with distinct posterior incisions; internal duct system visible through cuticle as elongated patches (Figs 8, 14); posterior end of first winding of internal duct system covered by LLs (Figs 9, 15).

##### Distribution.

Kuankuoshui Nature Reserve, Suiyang County, Guizhou Province, China.

##### Comments.

Males of *Pseudopoda acuminata* sp. n. could be included in the *Pseudopoda martensi*-group (Jäger, 2001). Males of *Pseudopoda martensi*-group are characterized by: embolus sickle shaped, strongly flattened, and arising in a prolateral position on the tegulum, first bending in a retrolateral direction and then running in a distal direction; small embolic projection present. Females are difficult to distinguish (Jäger 2001).

#### Pseudopoda emei sp. n.

http://zoobank.org/7ED92A2A-117E-4B10-ADB1-052398DA2D24

http://species-id.net/wiki/Pseudopoda_emei

Figs 18 –33

##### Type material.

**Holotype** ♂ (SP–SC–03–0050), from CHINA: Sichuan Province,Emei Mountain, Fuhu Temple (29°59'N, 103°48'E, 1800 m), 26.VII.2003, J.X. Zhang leg. (hand collecting), deposited in MHBU. **Paratype:** 1♂ (SP–SC–03–0051), 2♀♀ (SP–SC–03–0052–0053), same data as holotype; 1♀ (SP–SC–09–24), from CHINA: Sichuan Province,Emei Mountain, native forest, 24.IX.2010, Y.W. Zhao leg. (hand collecting), deposited in MHBU.

##### Etymology.

The specific name refers to the type locality, the mountain Emei; noun in apposition.

##### Diagnosis.

Males of *Pseudopoda emei* sp. n. resemble those of *Pseudopoda virgata* (Fox, 1936), *Pseudopoda kalinchoca* Jäger, 2001 and *Pseudopoda khimtensis* Jäger, 2001 by the strongly flattened embolus and long embolic tip, but can be distinguished by the following combination of characters: basal and middle part of embolus very broad, but with slender tip, embolic tip filiform, curving slightly upward (Figs 20–21, 27–28), prolateral margin of embolus with a small embolic projection, embolic projection shorter than 1/3 length of embolic tip (Figs 21, 28). Females can be distinguished from those of other *Pseudopoda* species by: posterior epigynal field wider than anterior part; anterior margin of the lateral lobes distinctly curved and pointing anterior-laterally (Figs 25, 30); lateral lobes large, with distinct ridges in dorsal view, the length of lateral margin of lateral lobes almost equal to that of median margin in dorsal view (Figs 26, 31); posterior half of first winding of internal duct system covered by lateral lobes (Figs 26, 31).

**Figures 18–21.**
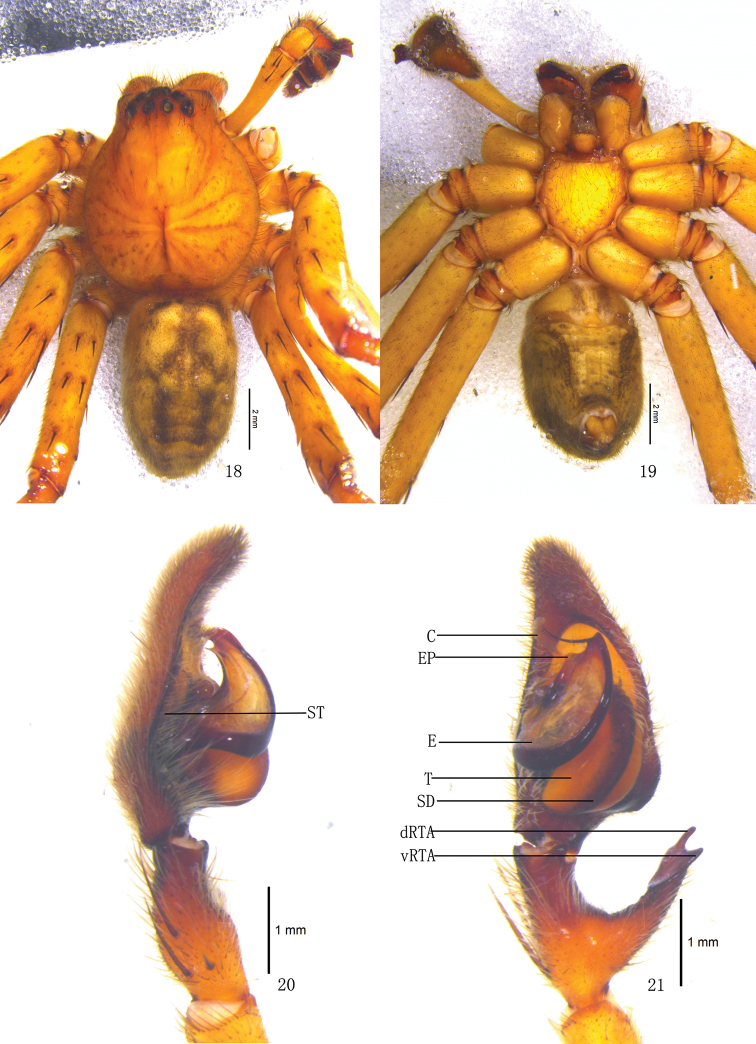
*Pseudopoda emei* sp. n., Male (SP–SC–03–0050): **18–19** Body (**18** dorsal **19** ventral) **20–21** Left palp (**20** prolateral **21** ventral). Abbreviations: C conductor; dRTA dorsal branch of retrolateral tibial apophysis; E embolus; EP embolic projection; SD sperm duct; ST subtegulum; T tegulum; vRTA ventral branch of retrolateral tibial apophysis. Scale bars: 2 mm (**18–19**); 1 mm (**20–21**).

**Figures 22–26.**
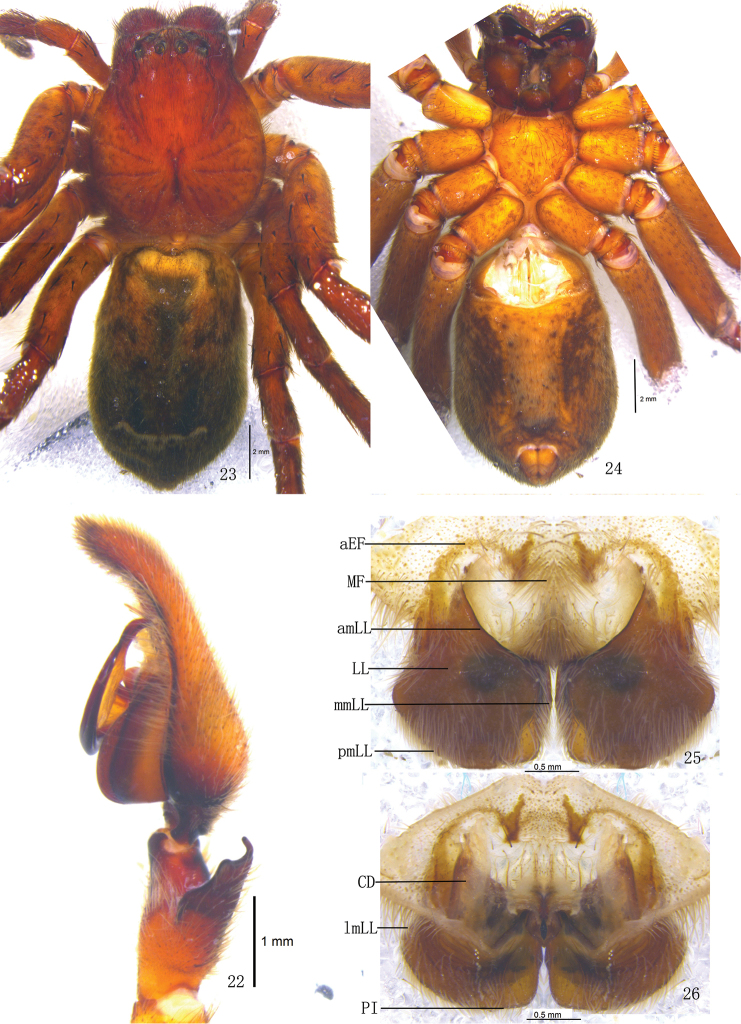
*Pseudopoda emei* sp. n., **22** Left palp of male (retrolateral). **23–26** Female (SP–SC–03–0052): **23–24** Body (**23** dorsal **24** ventral) **25–26** Epigyne (**25** ventral **26** dorsal). Abbreviations: aEF anterior margin of epigynal field; amLL anterior margin of lateral lobes; CD copulatory duct; LL lateral lobes of epigyne; lmLL lateral margin of lateral lobes; MF median field of epigyne; mmLL median margin of lateral lobes; pmLL posterior margins of lateral lobes; PI posterior incisions. Scale bars: 1 mm (**22**); 2 mm (**23–24**); 0.5 mm (**25–26**).

**Figures 27–33.**
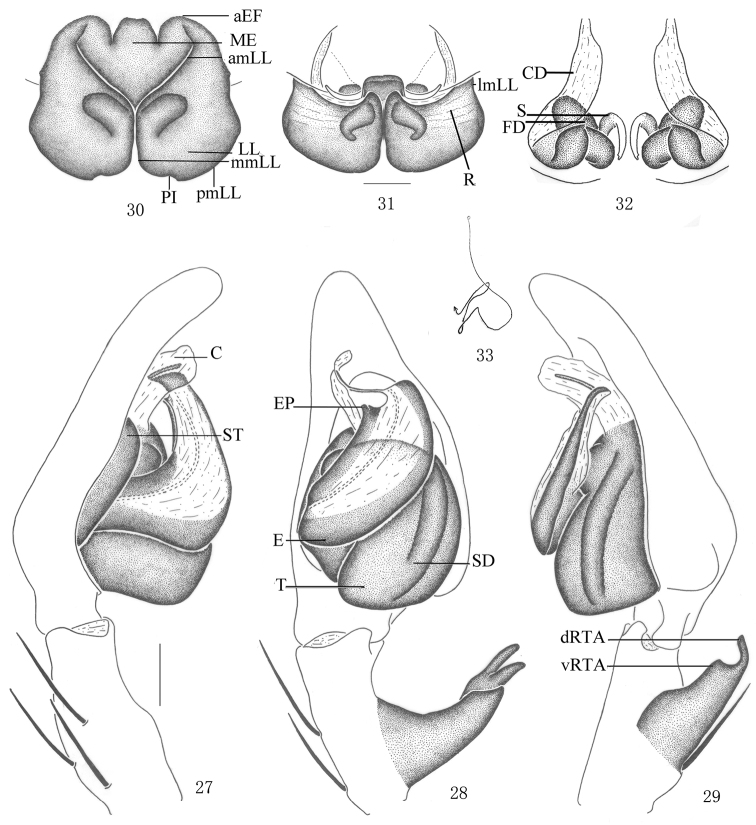
*Pseudopoda emei* sp. n., **27–29** Male (SP–SC–03–0050): Left palp (**27** prolateral **28** ventral **29** retrolateral). **30–33** Female (SP–SC–03–0052): **30–32** Epigyne (**30** ventral **31** dorsal **32** apical) **33** Schematic course of internal duct system, dorsal. Abbreviations: aEF anterior margin of epigynal field; amLL anterior margin of lateral lobes; C conductor; CD copulatory duct; dRTA dorsal branch of retrolateral tibial apophysis; E embolus; EP embolic projection; FD fertilization duct; LL lateral lobes of epigyne; lmLL lateral margin of lateral lobes; MF median field of epigyne; mmLL median margin of lateral lobes; pmLL posterior margins of lateral lobes; PI posterior incisions; R ridges; S spermathecae; SD sperm duct; ST subtegulum; T tegulum; vRTA ventral branch of retrolateral tibial apophysis. Scale bars: 0.5 mm.

##### Description.

Holotype (SP–SC–03–0050): total length 15.39; prosoma 7.21 long, 6.02 wide; opisthosoma 8.23 long, 4.82 wide. Prosoma with some setae. Fovea long, longitudinal. Coloration: Dorsal shield of prosoma yellow. Radial furrows and fovea dark brown. Chelicerae brown. Labium, gnathocoxae and sternum yellowish brown. Sternum with dark spots and setae. Legs yellow, with dark dots randomly distributed, and especially on the setae and spine bases of coxa and femur. Dorsal opisthosoma yellow, anterior part with black patches, cardiac pattern and muscle impressions dark brown, followed by three black transverse lines and two longitudinal black patches; lateral part with some smaller irregular patches (Fig. 18); venter yellow, with two black lateral lines and a black patch before spinnerets (Fig. 19). Both eye rows slightly recurved. Eye diameters and interdistances: AME 0.34, ALE 0.49, PME 0.36, PLE 0.47; AME–AME 0.21, AME–ALE 0.08, PME–PME 0.43, PME–PLE 0.52. MOA 1.17 long, anterior width 0.83, posterior width 1.16. Clypeus height 0.32. Chelicerae with three promarginal and four retromarginal teeth, and with thirteen denticles between them. Leg measurements: I 36.66 (9.51, 3.20, 10.22, 10.52, 3.21), II 36.85 (9.53, 3.23, 10.28, 10.56, 3.25), III 36.40 (9.43, 3.16, 10.19, 10.42, 3.20), IV 36.49 (9.48, 3.17, 10.22, 10.42, 3.20). Leg formula: 2143. Leg spination: palps 131, 101, 2121; femur I–III 323, IV 331; patella I–IV 101; tibia I–II 2226, III–IV 2126; metatarsus I–II 2024, III 2026, IV 3036. Male palp. Embolus long, arising from 8 o’clock-position on tegulum (Figs 20–21, 27–28), basal and middle part of embolus very broad, but with long and filiform tip, pointing ventro-prolaterally (Figs 20–21, 27–28); EP small (Figs 21, 28); sperm duct running submarginally along retrolateral margin of tegulum in ventral view (Figs 21–22, 28–29); RTA long, with broad base, arising medially to basally from tibia, dorsal branch narrow and curved, ventral branch short, wide, and as a small hump in retrolateral view (Figs 21–22, 28–29).

Females. Paratype (SP–SC–03–0052): total length 13.22; prosoma 6.41 long, 5.52 wide; opisthosoma 6.82 long, 4.89 wide. Coloration: Dorsal shield of prosoma reddish brown. Legs brown, with dark dots randomly distributed, and especially on the setae and spine bases of coxa and femur. Dorsal opisthosoma dark brown, cardiac pattern and muscle impressions black, followed by a transverse line composed of white hairs and two longitudinal black patches. Coloration pattern darker than male (Figs 23–24). Eye diameters and interdistances: AME 0.29, ALE 0.40, PME 0.33, PLE 0.39; AME–AME 0.29, AME–ALE 0.14, PME–PME 0.57, PME–PLE 0.47. MOA 1.17 long, anterior width 0.81, posterior width 1.14. Clypeus height 0.32. Leg measurements: I 22.27 (7.02, 2.83, 5.17, 5.17, 2.08), II 22.38 (7.06, 2.83, 5.18, 5.17, 2.14), III 21.71 (6.65, 2.81, 5.12, 5.15, 1.98), IV 21.89 (6.68, 2.83, 5.15, 5.19, 2.04). Leg formula: 2143. Leg spination: palps 131, 101, 2121, 2112; femur I–III 323, IV 321; patella I–IV 101; tibia I–IV 2126; metatarsus I–II 2024, III 2026, IV 3036. Epigyne. Epigynal field wider than long, anterior margin without longitudinal bands (Figs 25, 30); LLs width equal to length, touching each other along the median line, anterior margin of LLs distinctly curved and pointing anterior-laterally, posterior margins of LLs rounded, and with distinct posterior incisions (Figs 25, 30), LLs large, with distinct ridges in dorsal view (Figs 26, 31); internal duct system visible through cuticle as almost rectangular dark patches (Figs 25, 30); posterior end of first winding of internal duct system covered by LLs (Figs 26, 31).

##### Variation.

Male total body length from 15.32–15.39, and female from 13.22–14.21. Femur length of male: I from 9.48–9.51, II from 9.51–9.53, III from 9.42–9.43, IV from 9.46–9.48. Femur length of female: I from 7.02–7.05, II from 7.06–7.08, III from 6.65–6.66, IV from 6.68–6.70.

##### Distribution.

Emei Mountain, Sichuan Province, China.

##### Comments.

Males of *Pseudopoda emei* sp. n. could be included in the *Pseudopoda martensi*-group by: embolus sickle-shaped, strongly flattened, and arising in a prolateral position on the tegulum, first bending in a retrolateral direction and then running in a distal direction; small embolic projection present.

#### Pseudopoda lacrimosa sp. n.

http://zoobank.org/E5DF75DE-B3DD-40B9-80A8-4F9E93B0A72B

http://species-id.net/wiki/Pseudopoda_lacrimosa

Figs 34 –49

##### Type material.

**Holotype** ♂ (SP–GLGS–11–41), from CHINA: Yunnan Province, Fugong County, Maji Town, native forest (27°28'N, 98°51'E, 1700 m), 10.III.2011, Z.X. Li leg. (hand collecting), deposited in SWUC. **Paratype:** 1♀ (SP–GLGS–11–42), same data as holotype; 1♂ (SP–GLGS–11–23), 1♀ (SP–GLGS–11–24), from CHINA: Yunnan Province, Baoshan City, Tengchong County, Jietou Town, native forest (25°18'N, 98°21'E, 1850 m), 25.II.2011, L.Y. Wang leg. (hand collecting), deposited in SWUC.

##### Etymology.

The specific name is derived from the Latin word ‘lacrimosus, -a, -um’, meaning ‘lachrymal’, referring to the tear-drop shape of the epigynal median field; adjective.

##### Diagnosis.

Males of *Pseudopoda lacrimosa* sp. n. resemble those of *Pseudopoda everesta* Jäger, 2001 by the embolus with almost equal length of tip and projection, but can be distinguished by the following combination of characters: tip of embolus thin and long (Figs 37, 44); embolic projection large, strip-like (Figs 36–37, 43–44); tip of embolus and embolic projection pointed (Figs 37–38, 44–45); retrolateral tibial apophysis with a small tooth on anterior margin of ventral branch (Figs 37–38, 44–45). Females resemble those of *Pseudopoda diversipunctata* group by: anterior edges of lateral lobes oval and constrict; internal borders of lateral lobes not touching each other, but can be distinguished from other species of thisgroup by: median field of epigyne narrow, almost tear-drop-shaped (Figs 41, 46); lateral lobes almost as an oblique rectangle, anterior margins of lateral lobes distinctly curved, bracket shaped and pointing medially (Figs 42, 47).

**Figures 34–37.**
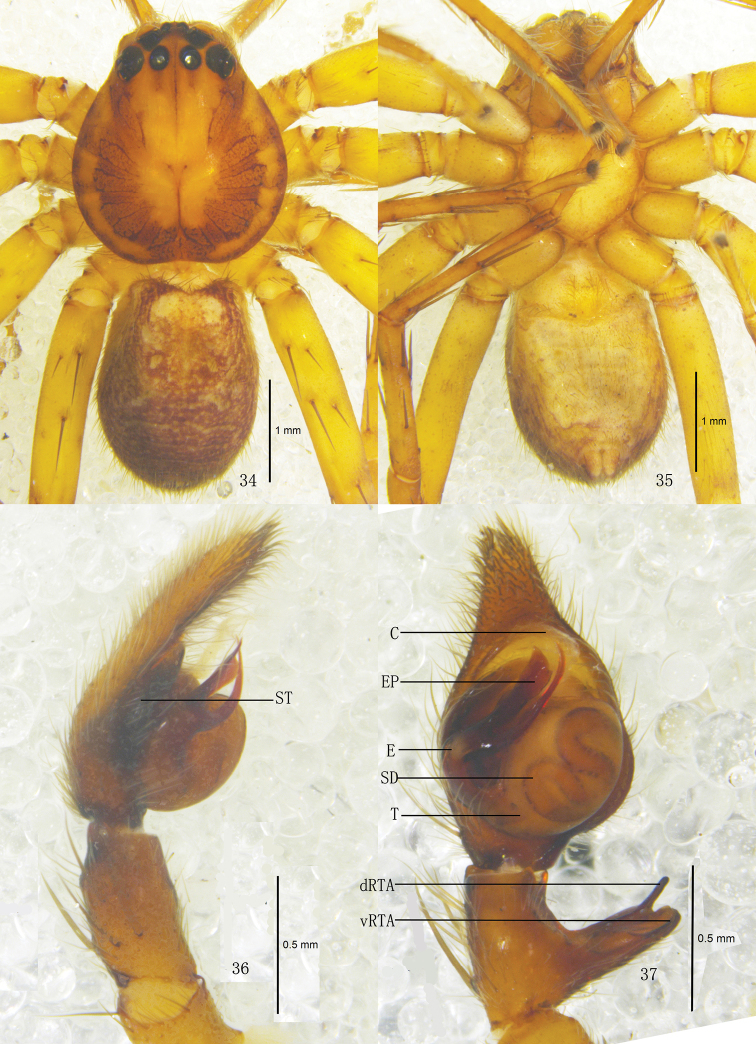
*Pseudopoda lacrimosa* sp. n., Male (SP–GLGS–11–41): **34–35** Body (**17** dorsal **18** ventral) **36–37** Left palp (**36** prolateral **37** ventral). Abbreviations: C conductor; dRTA dorsal branch of retrolateral tibial apophysis; E embolus; EP embolic projection; SD sperm duct; ST subtegulum; T tegulum; vRTA ventral branch of retrolateral tibial apophysis. Scale bars: 1 mm (**34–35**); 0.5 mm (**36–37**).

**Figures 38–42.**
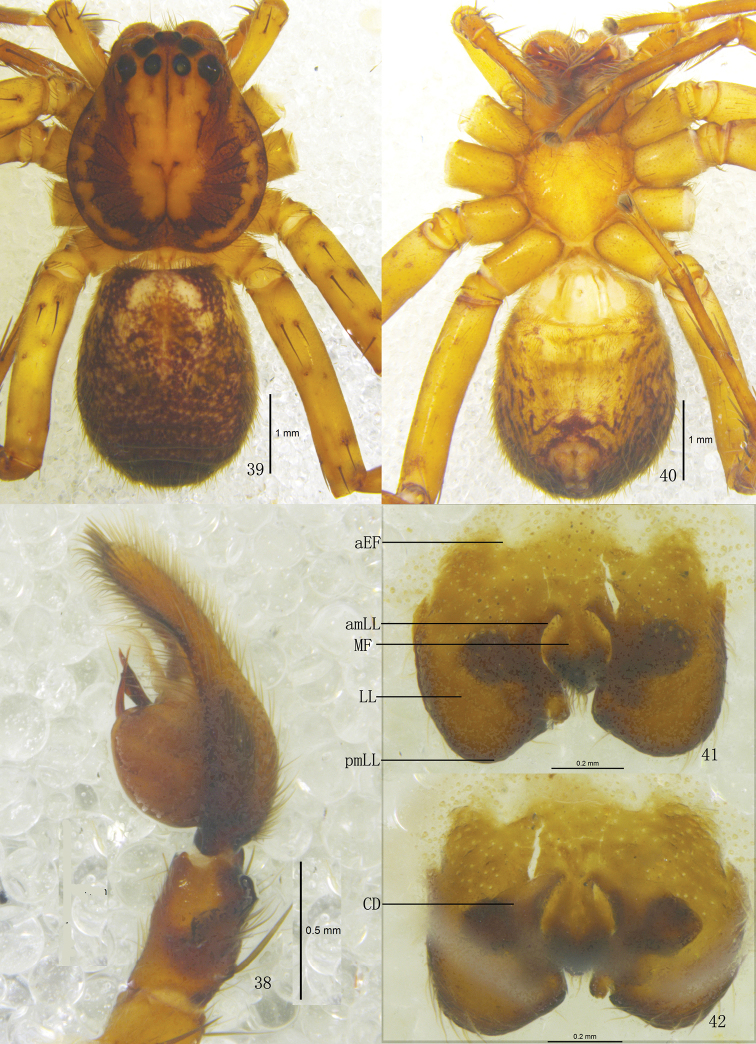
*Pseudopoda lacrimosa* sp. n., **38** Left palp of male (retrolateral). **39–42** Female (SP–GLGS–11–42): **39–40** Body (**39** dorsal **40** ventral) **41–42** Epigyne (**41** ventral **42** dorsal). Abbreviations: aEF margin of epigynal field; amLL anterior margin of lateral lobes; CD copulatory duct; LL lateral lobes of epigyne; MF median field of epigyne; pmLL posterior margins of lateral lobes. Scale bars: 1 mm (**39–40**); 0.5 mm (**38**); 0.2 mm (**41–42**).

**Figures 43–49.**
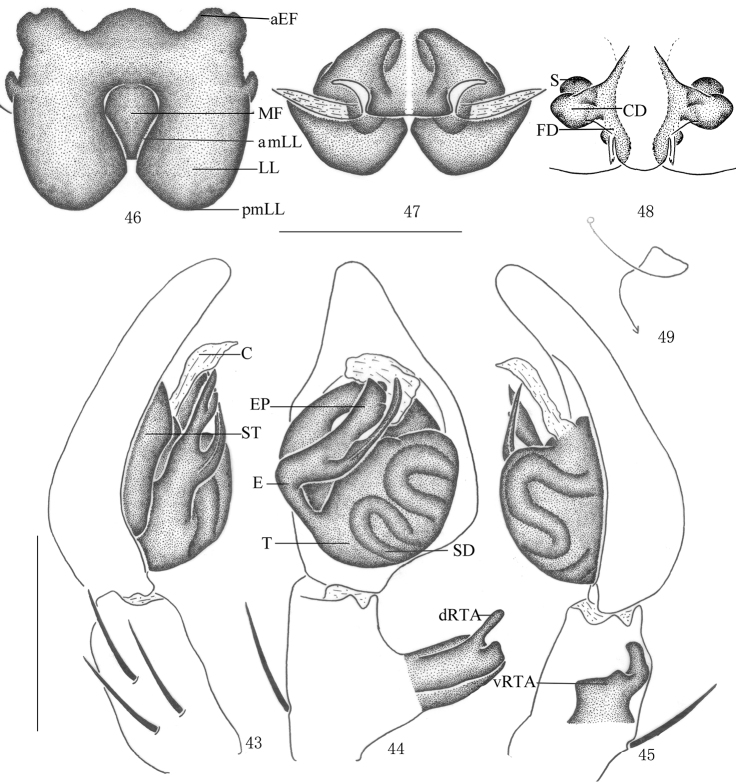
*Pseudopoda lacrimosa* sp. n., **43–45** Male (SP–GLGS–11–41): Left palp (**43** prolateral **44** ventral **45** retrolateral) **46–49** Female (SP–GLGS–11–42): **46–48** Epigyne (**46** ventral **47** dorsal **48** apical); **49** Schematic course of internal duct system, dorsa. Abbreviations: aEF anterior margin of epigynal field; amLL anterior margin of lateral lobes; C conductor; CD copulatory duct; dRTA dorsal branch of retrolateral tibial apophysis; E embolus; EP embolic projection; FD fertilization duct; LL lateral lobes of epigyne; MF median field of epigyne; pmLL posterior margins of lateral lobes; S spermathecae; SD sperm duct; ST subtegulum; T tegulum; vRTA ventral branch of retrolateral tibial apophysis. Scale bars: 0.5 mm.

##### Description.

Male. Holotype (SP–GLGS–11–41): total length 4.39; prosoma 2.11 long, 2.02 wide; opisthosoma 2.29 long, 1.43 wide. Fovea long, longitudinal. Coloration: Dorsal borders of prosoma brown, as the thick U-shaped pattern, rest yellow. Radial furrows and fovea dark brown. Chelicerae, labium, gnathocoxae and sternum yellow. Sternum with dark setae. Legs yellow, with dark dots randomly distributed, and especially on the setae and spine bases of femur. Dorsal opisthosoma dark brown, with some small pale spots distributed around the opisthosoma, anterior part with two pale patches, cardiac pattern brown, muscle impressions yellow (Fig. 34); venter yellow (Fig. 35). Both eye rows slightly recurved. Eye diameters and interdistances: AME 0.13, ALE 0.26, PME 0.18, PLE 0.27; AME–AME 0.10, AME–ALE 0.03, PME–PME 0.16, PME–PLE 0.23. MOA 0.52 long, anterior width 0.30, posterior width 0.55. Clypeus height 0.21. Chelicerae with three promarginal and four retromarginal teeth, with eight denticles between them. Leg measurements: I 11.04 (2.41, 1.60, 2.81, 2.72, 1.50), II 12.24 (2.43, 1.64, 2.88, 2.73, 1.56), III 10.86 (2.33, 1.62, 2.69, 2.75, 1.47), IV 10.97 (2.38, 1.60, 2.78, 2.72, 1.49). Leg formula: 2143. Leg spination: palps 131, 101, 2121; femur I 223, II 323, III 322, IV 331; patella I–IV 001; tibia I–III 2026, IV 2126; metatarsus I–II 0004, III 2026, IV 3036. Male palp. Tegulum large (Figs 37, 44); embolus with thin tip and arising from 9 o’clock-position on tegulum (Figs 36–37, 43–44); embolic projection long, strip-like (Figs 36–37, 43–44); sperm duct S-shaped, running retrolaterally in the tegulum (Figs 37, 44–45); RTA with broad base, arising medially from tibia, dorsal branch thin, slightly curved, longer than ventral branch, ventral branch wide and with a small tooth on anterior margin (Figs 37–38, 44–45).

Females. Paratype (SP–GLGS–11–42): total length 6.51; prosoma 3.11 long, 2.22 wide; opisthosoma 3.42 long, 2.12 wide. Coloration: Venter yellow, with a black patch before spinnerets and some small black spots distributed laterally (Fig. 40). Shape, color and markings of body as in male (Figs 39–40). Eye diameters and interdistances: AME 0.13, ALE 0.26, PME 0.21, PLE 0.30; AME–AME 0.14, AME–ALE 0.07, PME–PME 0.27, PME–PLE 0.30. MOA 0.64 long, anterior width 0.39, posterior width 0.61. Clypeus height 0.21. Leg measurements: I 12.10 (3.82, 1.23, 2.81, 2.61, 1.63), II 12.21 (3.86, 1.23, 2.88, 2.63, 1.61), III 11.55 (3.69, 1.17, 2.62, 2.55, 1.52), IV 11.94 (3.78, 1.19, 2.78, 2.59, 1.60). Leg formula: 2143. Leg spination: palps 131, 101, 2121, 2112; femur I–II 323, III 322, IV 331; patella I–IV 001; tibia I–III 2026, IV 2126; metatarsus I–II 0004, III 2026, IV 3036. Epigyne. Median field of epigyne narrow, almost oval, anterior margin distinct, without longitudinal bands (Figs 41, 46); LLs longer than wide, closer to each other at the anterior median line, anterior and posterior margins of the LLs distinctly curved, anterior margins bracket shaped, (Figs 41, 46); posterior part of first winding of internal duct system wider than anterior part (Figs 42, 47).

##### Variation.

Male total body length from 4.36–4.39, and female from 6.45–6.51. Femur length of male: I from 2.38–2.41, II from 2.41–2.43, III from 2.32–2.33, IV from 2.36–2.38. Femur length of female: I from 3.80–3.82, II from 3.85–3.86, III from 3.65–3.69, IV from 3.76–3.78.

##### Distribution.

Maji Town, Fugong County, Yunnan Province, China; Jietou Town, Tengchong County, Baoshan City, Yunnan Province, China.

##### Comments.

Females of *Pseudopoda lacrimosa* sp. n. could be included in the *Pseudopoda diversipunctata*-group (Jäger, 2001). Females of this group are characterized by: lateral lobes of epigyne touching each other only at posterior part, the first winding of internal duct system running from laterally to the median line and the loop situated ventrally (Jäger 2001). On the other hand, males of *Pseudopoda lacrimosa* sp. n. have long embolic projection and tip, which could place them in the *Pseudopoda latembola*-group (Jäger, 2001).

#### Pseudopoda robusta sp. n.

http://zoobank.org/A77AD9CF-DD57-476B-B402-1C1EDF23C0C3

http://species-id.net/wiki/Pseudopoda_robusta

Figs 50 –65

##### Type material.

**Holotype** ♂ (SP–CQ–08–26), from CHINA: Chongqing Municipality, Jinyun Mountain, native forest, (29°49'N, 106°21'E, 1600 m), 26.IV.2008, Z.S. Zhang leg. (hand collecting), deposited in SWUC.Paratype: 1♂ (SP–CQ–08–27), 4♀♀ (SP–CQ–08–28–31), same data as holotype.

##### Etymology.

The specific name is derived from the Latin word ‘robustus, -a, -um’, meaning ‘strong’, referring to the robust retrolateral tibial apophysis; adjective.

##### Diagnosis.

Males of *Pseudopoda robusta* sp. n. resemble those of *Pseudopoda sinapophysis* Jäger & Vedel, 2007 by the simple embolus conformation, but can be distinguished by the following combination of characters: embolus large and long, flagelliform (Figs 52–53, 59–60); RTA massive in ventral view, with blunt tip (Figs 53–54, 60–61). Females of *Pseudopoda robusta* sp. n. resemble those of *Pseudopoda diversipunctata* group by: lateral lobes of epigyne touching each other only at posterior part; anterior edges of lateral lobes constrict, but can be distinguished from other species of thisgroup by: median field of epigyne wider than long, distinctly U-shaped (Figs 57, 62); anterior margin of the LLs pointing anteriorly (Figs 57, 62); internal duct system with visible lateral loops in dorsal view (Figs 58, 63–64), the first winding wide, its length twice its width (Figs 58, 63–64).

**Figures 50–53.**
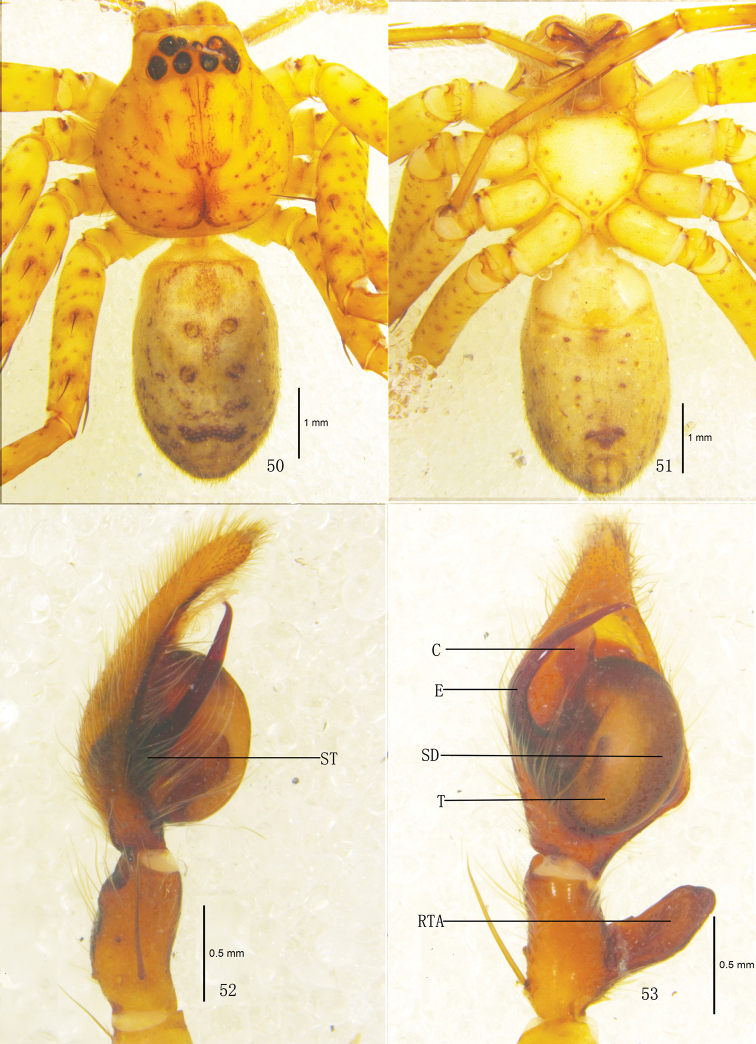
*Pseudopoda robusta* sp. n.,Male (SP–CQ–08–26): **50–51** Body (**50** dorsal **51** ventral) **52–53** Left palp (**52** prolateral **53** ventral). Abbreviations: E embolus; EP embolic projection; RTA retrolateral tibial apophysis; SD sperm duct; ST subtegulum; T tegulum. Scale bars: 1 mm (**50–51**); 0.5 mm (**52–53**).

**Figures 54–58.**
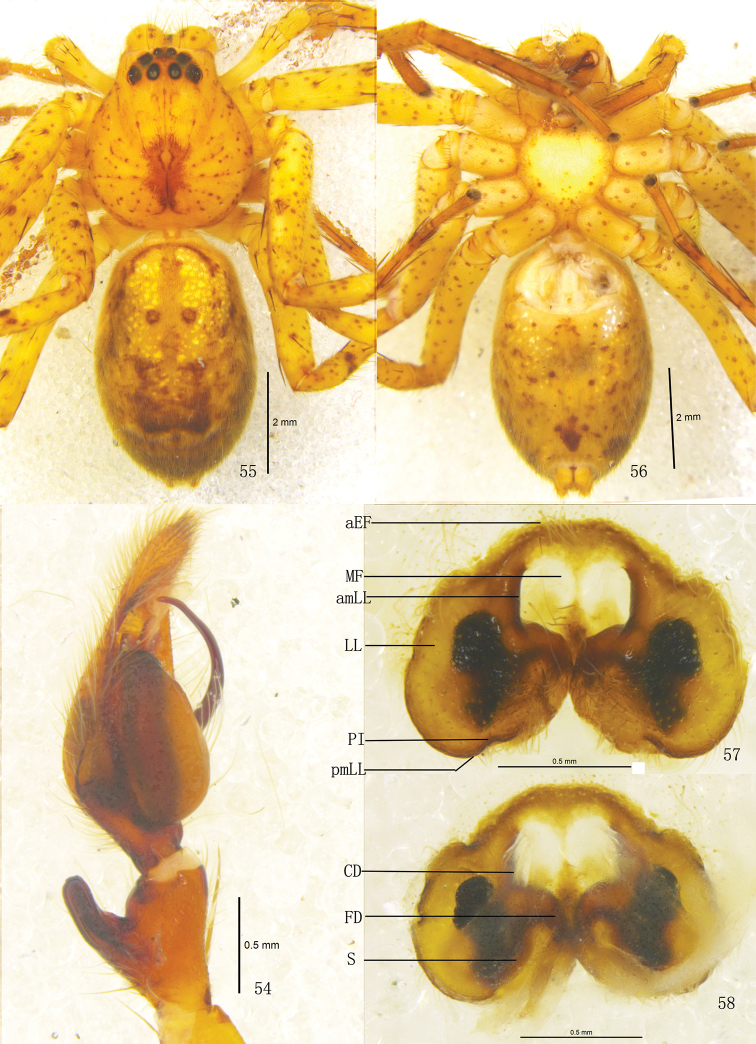
*Pseudopoda robusta* sp. n., **54** Left palp of male (retrolateral). **55–58** Female (SP–CQ–08–28): **55–56** Body (**55** dorsal **56** ventral) **57–58** Epigyne (**57** ventral **58** dorsal). Abbreviations: aEF margin of epigynal field; amLL anterior margin of lateral lobes; CD copulatory duct; FD fertilization duct; LL lateral lobes of epigyne; MF median field of epigyne; pmLL posterior margins of lateral lobes; PI posterior incisions; S spermathecae; Scale bars: 2 mm (**55–56**); 0.5 mm (**54**, **57–58**).

**Figures 59–65.**
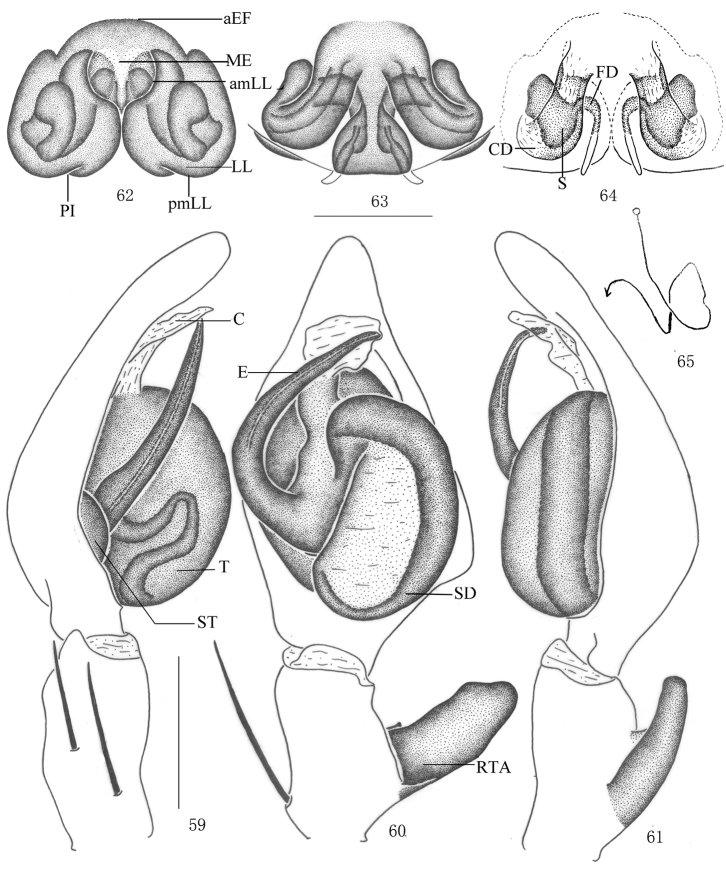
*Pseudopoda robusta* sp. n., **59–61** Male (SP–CQ–08–26): Left palp (**59** prolateral **60** ventral **61** retrolateral) **62–65** Female (SP–CQ–08–28): **62–64** Epigyne (**62** ventral **63** dorsal **64** apical) **65** Schematic course of internal duct system, dorsa. Abbreviations: aEF anterior margin of epigynal field; amLL anterior margin of lateral lobes; C conductor; CD copulatory duct; E embolus; EP embolic projection; FD fertilization duct; LL lateral lobes of epigyne; MF median field of epigyne; pmLL posterior margins of lateral lobes; RTA retrolateral tibial apophysis; S spermathecae; SD sperm duct; ST subtegulum; T tegulum. Scale bars: 0.5 mm.

##### Description.

Male. Holotype (SP–CQ–08–26): total length 6.90; prosoma 3.21 long, 3.02 wide; opisthosoma 3.73 long, 2.12 wide. Fovea long, longitudinal. Coloration: Dorsal shield of prosoma yellow, margin with reddish brown patches. Radial furrows and fovea reddish brown, fovea surrounded with reddish brown patch. Ocular area brown. Chelicerae, labium, gnathocoxae and sternum yellow. Sternum with dark spots and setae. Legs yellow, with dark dots randomly distributed, and especially on the setae and spine bases of coxa, femur, patella and tibia. Dorsal opisthosoma yellow, anterior part with many smallwhite patches, cardiac pattern yellowish brown, muscle impressions dark brown, followed by two longitudinal black patches and a black transverse bar, and with some dark brown patches laterally (Fig. 50); venter yellow, with small black patches and a black patch before spinnerets (Fig. 51). Both eye rows slightly recurved. Eye diameters and interdistances: AME 0.13, ALE 0.29, PME 0.18, PLE 0.26; AME–AME 0.10, AME–ALE 0.04, PME–PME 0.18, PME–PLE 0.29. MOA 0.62 long, anterior width 0.39, posterior width 0.57. Clypeus height 0.23. Chelicerae with three promarginal and four retromarginal teeth, with eleven denticles between them. Leg measurements: I 14.14 (5.01, 1.40, 3.22, 2.82, 1.69), II 15.07 (5.23, 1.43, 3.18, 2.86, 1.75), III 13.30 (4.73, 1.26, 2.89, 2.72, 1.70), IV 13.99 (4.98, 1.37, 3.22, 2.72, 1.70). Leg formula: 2143. Leg spination: palps 131, 101, 2121; femur I–III 323, IV 331; patella I–III 001, IV 000; tibia I 2026, II–IV 2126; metatarsus I–II 2024, III 2026, IV 3036. Male palp. Tegulum almost oval (Figs 53, 60); embolus wide, flagelliform, arising from 9-o’clock-position on tegulum, its tip pointing retrolaterally (Figs 52–53, 59–60); sperm duct inverted C-shaped, running along retrolateral margin of tegulum (Figs 53, 60); RTA strong, rod-like, arising medially from tibia, with blunt tip (Figs 53–54, 60–61).

Females. Paratype (SP–CQ–08–28): total length 7.40; prosoma 3.61 long, 3.22 wide; opisthosoma 3.82 long, 2.72 wide. Color and markings of body lighter than in male (Figs 55–56). Eye diameters and interdistances: AME 0.18, ALE 0.31, PME 0.23, PLE 0.30; AME–AME 0.16, AME–ALE 0.05, PME–PME 0.23, PME–PLE 0.34. MOA 0.68 long, anterior width 0.51, posterior width 0.73. Clypeus height 0.23. Leg measurements: I 12.08 (3.72, 1.23, 2.81, 3.09, 1.23), II 12.28 (3.76, 1.23, 2.98, 3.03, 1.28), III 11.53 (3.65, 1.21, 2.62, 2.85, 1.20), IV 11.85 (3.68, 1.23, 2.75, 2.99, 1.20). Leg formula: 2143. Leg spination: palps 131, 101, 2121, 2112; femur I–II 323, III 322, IV 331; patella I–III 001, IV 000; tibia I–II 2026, III–IV 2126; metatarsus I–II 2024, III 2026, IV 3036. Epigyne. Median field of epigyne distinctly U-shaped, anterior margin distinct, without longitudinal bands (Figs 57, 62); width of LLs equal to length, touching each other only slightly, posterior margins of LLs with distinct posterior incisions (Figs 57, 62); posterior end of first winding of internal duct system freely visible, spermathecae situated ventrally, space between fertilization duct and first winding smaller than width of first winding (Figs 58, 63).

##### Variation.

Male total body length from 6.90–6.96, and female from 7.32–7.40. Femur length of male: I from 5.01–5.04, II from 5.23–5.24, III from 4.73–4.75, IV from 4.98–4.99. Femur length of female: I from 3.70–3.72, II from 3.75–3.76, III from 3.64–3.65, IV from 3.66–3.768.

##### Distribution.

Jinyun Mountain, Chongqing Municipality, China, type locality.

##### Comments.

Females of *Pseudopoda robusta* sp. n. could be included in the *Pseudopoda diversipunctata*-group by: lateral lobes of epigyne touching each other only at posterior part. On the other hand, males of *Pseudopoda robusta* have simple embolus conformation, which could be considered really different and not similar to any group.

**Figure 66.**
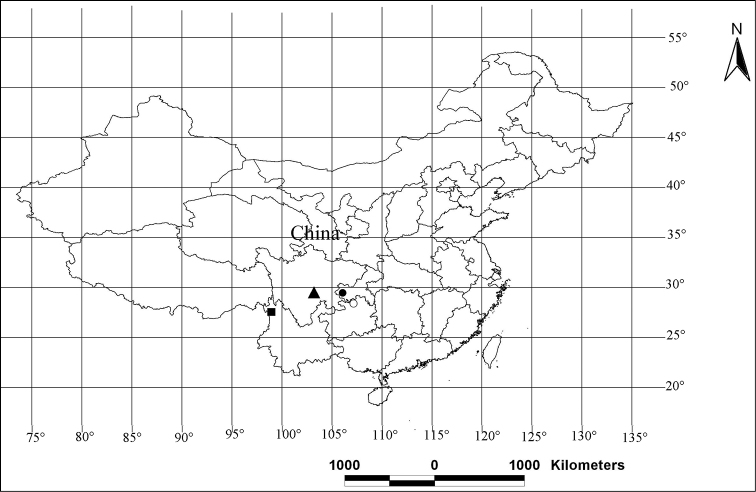
Distribution patterns of the new species of the genus *Pseudopoda* in China. ○ *Pseudopoda acuminata*; ▲ *Pseudopoda emei*; ■ *Pseudopoda lacrimosa*; ● *Pseudopoda robusta*.

## Supplementary Material

XML Treatment for Pseudopoda

XML Treatment for Pseudopoda acuminata

XML Treatment for Pseudopoda emei

XML Treatment for Pseudopoda lacrimosa

XML Treatment for Pseudopoda robusta
